# Can quality of recovery be enhanced by premedication with midazolam? A prospective, randomized, double-blind study in females undergoing breast surgery

**DOI:** 10.1097/MD.0000000000006107

**Published:** 2017-02-17

**Authors:** Myoung Hwa Kim, Min Soo Kim, Jae Hoon Lee, Jae Hi Seo, Jeong-Rim Lee

**Affiliations:** a Department of Anesthesiology and Pain Medicine; b Anesthesia and Pain Research Institute, Yonsei University College of Medicine, Yonsei-ro 50-1, Seodaemun-gu, Seoul, Republic of Korea.

**Keywords:** mastectomy, midazolam, premedication, quality of recovery

## Abstract

**Background:**

Preoperative anxiety is known to be related with the postoperative outcomes, although it remains unclear whether pharmacologic anxiolysis preoperatively leads to better postanesthesia recovery. Hence, the purpose of this study was to assess whether midazolam premedication would result in improved Quality of Recovery-40 survey scores, as a postoperative recovery parameter, in female patients undergoing mastectomy.

**Methods:**

This randomized double-blind study was performed at Severance Hospital, Yonsei University Health System, Seoul, Republic of Korea. Eighty-two females undergoing breast cancer surgery with propofol-remifentanil anesthesia were enrolled and randomized to receive midazolam 0.02 mg kg^−1^ (group M) or saline (group C). Anesthesia was conducted with total intravenous anesthesia using propofol and remifentanil. On postoperative day 1, the Quality of Recovery-40 survey scores were surveyed.

**Results:**

The global Quality of Recovery-40 survey scores on postoperative day 1 did not significantly differ between groups M and C (183 vs 181, *P* = 0.568). However, the induction time was significantly shorter in group M (3.2 vs 4.5 min, *P* < 0.001), as was the total intraoperative propofol consumption (705 vs 1004 mg; *P* = 0.022).

**Conclusion:**

Midazolam premedication does not seem to improve the postoperative quality of recovery, though group M showed faster induction and less propofol consumption.

## 1 Introduction

It is well known that high levels of preoperative stress or anxiety negatively impacts postoperative recovery.^[1]^ Meanwhile, premedication is commonly used for patients experiencing anxiety before anesthesia and surgery,^[2]^ with midazolam considered 1 reliable premedication drug because of its short-acting, dependable effects of sedation, anxiolysis, and meaningful retrograde amnesia, with few adverse effects.^[3]^ Therefore, anxiolysis with midazolam premedication can be assumed to result in better functional recovery after anesthesia and surgery, although this assumption has not been fully examined.^[4,5]^

Regarding the evaluation of postoperative functional recovery, the most widely used method is the Quality of Recovery 40 (QoR-40) survey. The high test–retest reliability, internal consistency, and split-half coefficient of the QoR-40 have led to its recognition as a useful and practical survey method, and its use has been validated for various patients undergoing diverse surgical procedures.^[6]^ Moreover, the effects of some particular premedication drugs such as dexamethasone and gabapentin have also been evaluated by the QoR-40, though none of these drugs aim at anxiety reduction.^[7–9]^ In fact, research on recovery scores associated with anxiolytics is scarce.

Herein, we hypothesized that midazolam premedication for anxiolysis before anesthesia would affect postanesthesia recovery. Accordingly, females undergoing breast cancer surgery with propofol-remifentanil anesthesia were enrolled, and we aimed to compare the QoR-40 as a postoperative recovery parameter between patients premedicated with midazolam and those administered saline.

## 2 Methods

This prospective randomized double-blind study was performed from September 2013–August 2014 at Severance Hospital, Yonsei University Health System, Seoul, Republic of Korea. The protocol was approved by the institutional review board (IRB number 4-2013-0444) and the study was registered as a clinical trial (NCT01945476).

### 2.1 Study population

Written informed consent was obtained from all patients before enrolment. Females aged 20 to 65 years with an American Society of Anesthesiologists physical status of 1–2, scheduled to undergo elective partial or total mastectomy under general anesthesia were considered eligible. Patients taking medications with central nervous system effects, such as sedatives and sleeping pills, and those who reported drinking more than 1 bottle of alcohol/day were excluded. Additional exclusion criteria were body mass index >30 kg m^−2^, known propofol allergy, and simultaneously scheduled autologous muscle reconstruction surgery.

### 2.2 Study design

We conducted a randomized, parallel group, double-blind study. The patients were randomly allocated to the midazolam premedication group (group M) or the control (NaCl 0.9%) group (group C) on the morning of the operation using a random number generator (http://www.random.org/). The ratio of allocation was 1:1. The assignments were concealed in a sealed envelope; the randomization was not blocked or stratified. Patients in group M received 0.02 mg kg^−1^ midazolam in 5 mL normal saline and those in group C received an equivalent volume (5 mL) of saline only. The researcher, who did not participate in the conduction of anesthesia or the postanesthesia surveys, prepared the study drugs. All study drugs were labeled as “premedication drug” to ensure blinding of the attending anesthesiologists, the investigator who administered the QoR-40 survey, and the patients.

### 2.3 Procedure and intervention

The study drugs were administered 30 minutes before entering the operating room, and the patients were monitored for respiratory rate and peripheral oxygen saturation in the pretreatment room immediately after administration. Supplemental oxygen was provided if a patient's respiratory rate was <8 breaths min^−1^ or if the peripheral oxygen saturation was <92%.

Upon arrival to the operating room, routine monitoring, including electrocardiography, pulse oximetry, noninvasive blood pressure, bispectral index (BIS) monitoring, and capnography were applied. All patients received total intravenous anesthesia (TIVA) with an effect-site target controlled infusion (TCI) of propofol and remifentanil, which was prepared with 2% propofol (Fresofol 2% injection 50 mL vial; Fresenius Kabi, Austria) and 20 μg mL^−1^ remifentanil (Ultiva injection 1 mg vial; GlaxoSmithKline, Belgium). A commercially available TCI pump (Orchestra Base Primea; Fresenius Vial, France) was used. The effect-site TCI for propofol was based on Schnider's pharmacokinetic model and that for remifentanil on the model by Minto and colleagues.^[10,11]^ Anesthesia was induced with remifentanil 3 μg mL^−1^ followed by propofol 3 μg mL^−1^ initially, and the propofol concentration was adjusted every 30 seconds until the patient lost consciousness, after which 0.6 mg kg^−1^ rocuronium was administered to facilitate intubation. Tracheal intubation was performed in all patients using a 6.5-mm (internal diameter) tracheal tube, and the cuff pressure was maintained at 20 to 25 cmH_2_O. Mechanical ventilation was maintained with a tidal volume of 8 mL kg^−1^ ideal body weight, and the ventilator frequency was adjusted to maintain an end-tidal carbon dioxide concentration of 35 to 40 mm Hg using an air/oxygen mixture (fraction of inspired oxygen, 0.5). The body temperature was maintained at 36 to 37°C. During the surgical procedure, propofol was titrated to maintain BIS values of 40 to 60 and remifentanil was adjusted to keep the blood pressure and heart rate within 20% of the baseline values. Approximately 30 minutes before operation completion, propacetamol 20 mg kg^−1^ was administered over 10 minutes, and 1 μg kg^−1^ of fentanyl and 0.3 mg ramosetron were injected approximately 15 minutes before the end of the surgery. The propofol and remifentanil infusions were discontinued upon surgery completion, and the patients were administered 30 μg kg^−1^ of neostigmine with 6 μg kg^−1^ of glycopyrrolate to reverse any residual neuromuscular blockade. When consciousness and spontaneous respiration were adequately restored, the endotracheal tube was removed and the patient was transferred to the post anesthesia recovery unit (PACU). The patients were transferred to the ward after at least 30 minutes in the PACU when they fulfilled the discharge criteria according to the modified Aldrete scoring system (score ≥ 9, with no score of 1 in any individual category).^[12]^

### 2.4 Assessment of outcomes

#### 2.4.1 Primary outcomes

A single researcher who was unaware of the group assignments visited each patient before the surgery and on postoperative day 1 (POD 1), between 6 and 8 pm, to conduct the QoR-40 surveys. The global QoR-40 score on POD 1 was the primary endpoint of this study. The QoR-40 measures 5 general quality of life dimensions: physical comfort (12 items), emotional state (9 items), physical independence (5 items), psychological support (7 items), and pain (7 items). Each item is graded on a 5-point Likert scale, and the global score ranges from 40 (extremely poor quality of recovery) to 200 (excellent quality of recovery). The QoR-40 scoring system was explained in detail to all participants and the questionnaire was completed in the presence of the researcher and reviewed to ensure accurate comprehension of all questions.

#### 2.4.2 Secondary outcomes

The sedation scores (1–5) were recorded in the pretreatment room and PACU. Sedation scores of 1, 2, 3, 4, and 5 indicated that the patient was completely awake, partly asleep, responsive to vocal commands but seemed asleep, showed signs of sleeping and reacted to vocal commands more slowly, and seemed to be asleep and did not react to vocal commands, but did react to stimulation, respectively.^[13]^

During anesthesia induction, we recorded the effect-site concentration and total infused amount of propofol required and the times to loss of consciousness and BIS < 60. The total amount of propofol and remifentanil infused during anesthesia, and the surgical and anesthesia durations were also recorded.

To determine the recovery time from anesthesia, we measured the effect-site concentration of propofol and the time from stopping the anesthetic infusion to response to verbal commands, as well as the effect-site concentration of propofol and the time to removal of the endotracheal tube.

During the stay in the PACU, we used the numeric rating scale with a 0 (no pain) to 10 (worst pain imaginable) scoring system to evaluate the immediate postoperative pain. We recorded the pain scores, nausea, and vomiting events, and any other symptoms reported by the patients as well as any medications that were given to relieve the symptoms. Once the patients were in a ward, we recorded the analgesic requirements during the initial 24 postoperative hours, occurrence of nausea and/or vomiting, total antiemetic requirement, and length of hospital stay.

### 2.5 Statistical analysis

Considering a number of previous reports that regarded an average difference of ≥10 in the QoR-40 scores as significant and that demonstrated that women who undergo general anesthesia scored an average of 162 on the QoR-40, along with an alpha of 0.05, and power of 90%,^[6,14]^ we determined that 34 patients per group were required. Therefore, considering a case loss of 20%, we planned to collect data from 41 patients per group.

Data analysis was based on the intent-to-treat approach. We tested every hypothesis at a 5% significance level and principally conducted a dual-examination. Shapiro-Wilk and Kolmogorov–Smirnov tests were performed to assess the hypothesis of normal distribution. All continuous variables are expressed as the mean (± standard deviation) or median (interquartile range), and all nominal factors as n (proportion, %). We performed Student's *t*-test or the Mann–Whitney *U* test for inter-group comparisons of the QoR-40 scores and other continuous variables. Additionally, the paired *t*-test or Wilcoxon signed rank test was used to compare the intra-group differences in the global and dimension QoR-40 scores preoperatively and on POD 1. Nominal variables were compared using the Chi-square and Fisher's exact tests. We used SPSS version 23 (SPSS, Inc., Chicago, IL) for the statistical analyses, with *P* < 0.05 indicating statistical significance.

## 3 Results

### 3.1 Study population

A total of 91 patients were assessed for eligibility, of whom 82 patients were included. Among them, 1 patient was withdrawn due to a change in the surgical plan during operation. Thus, we finally collected and analyzed data from 81 patients within hospital stay. And, there were no significant differences between the groups in terms of the patient characteristics (Table 1).

**Table 1:**
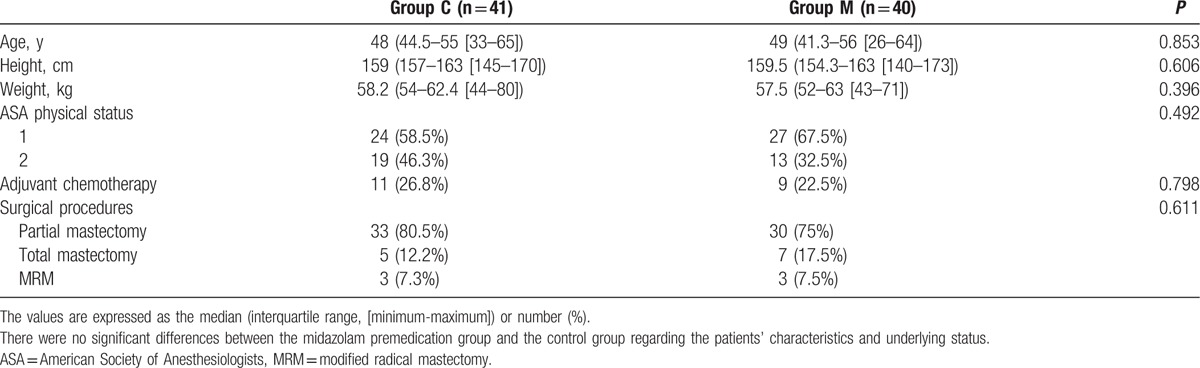
Characteristics of the study patients.

### 3.2 Primary outcome

Table 2 shows the global and dimensional (emotional state, physical comfort, psychological support, physical independence, and pain) QoR-40 scores on POD 1. The baseline preoperative global and dimensional QoR-40 scores did not differ significantly between groups M and C (179 vs 180, *P* *=* 0.784), nor were there any significant differences in the global and dimensional QoR-40 scores on POD1 (181 vs 183, *P* = 0.568). The subscores for the QoR-40 dimensions of emotional state and psychological support were improved on POD1 compared with the preoperative values in both study groups according to the intra-group comparison.

**Table 2:**
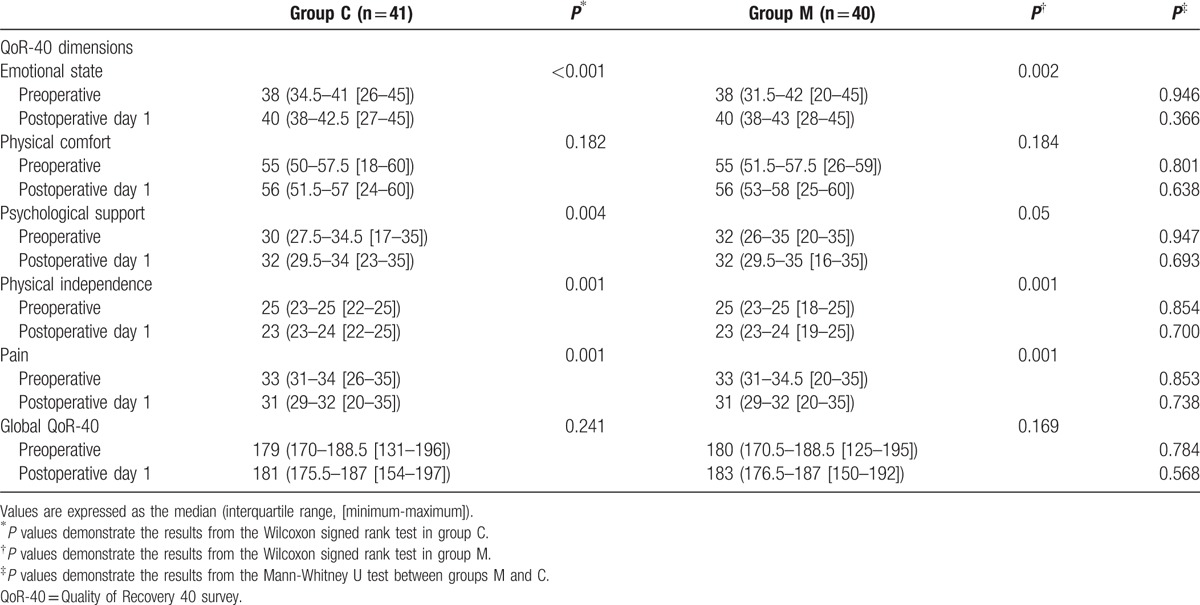
The global and dimensional QoR-40 scores.

### 3.3 Secondary outcome

Table 3 presents the perioperative data. The sedation score was significantly higher before induction (2 vs 1), the time to loss of consciousness was faster (1.5 vs 3.3 s), and loss of consciousness was achieved at a lower concentration (3 vs 4.1 μg) and dose (58.9 vs 96 mg) of propofol in group M compared with in group C. Further, the intraoperative propofol consumption was lower in group M compared with in group C (705 vs 1004 mg, *P* = 0.022).

**Table 3:**
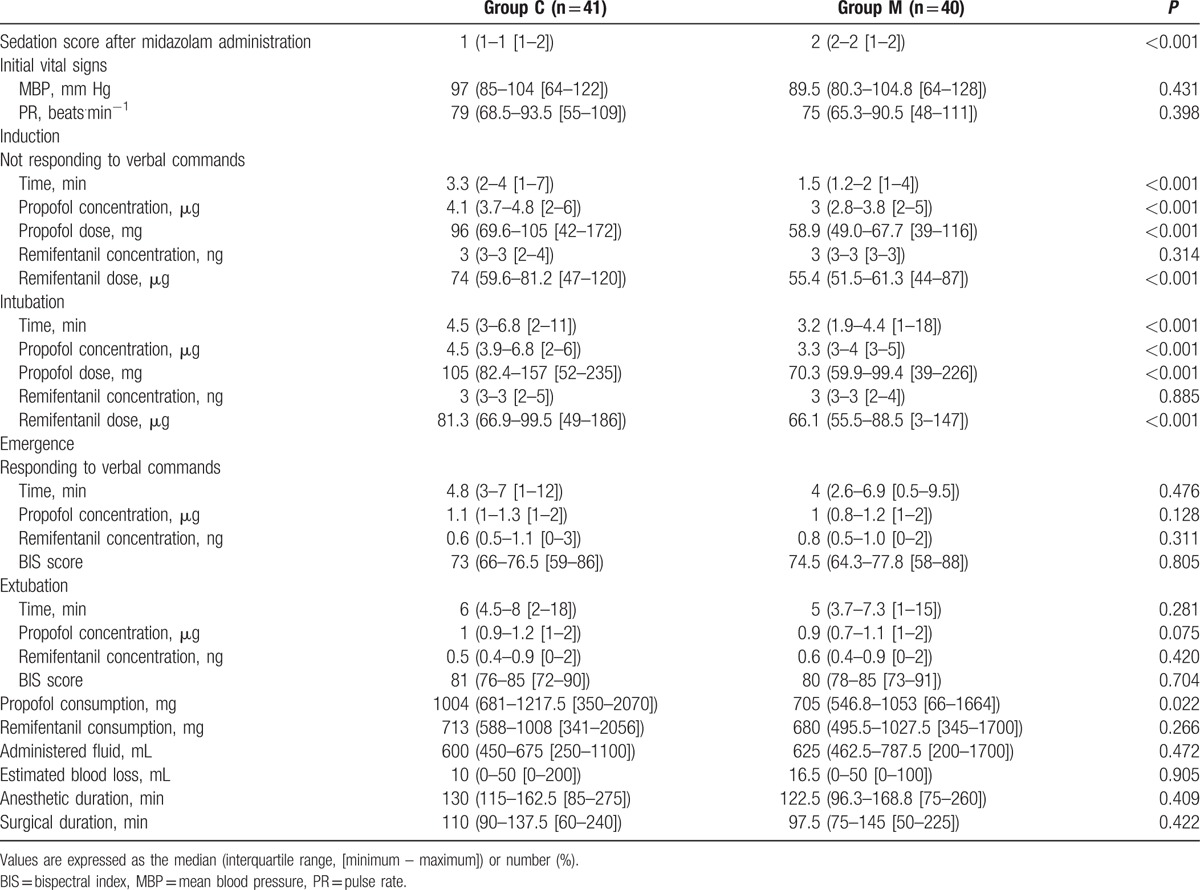
Perioperative data.

The postoperative recovery parameters examined in the PACU are described in Table 4. The sedation scores did not significantly differ between the groups at admission or at discharge from the PACU. The pain scores were significantly lower in group M at discharge from PACU (*P* = 0.004), though there were no significant differences in terms of analgesic requirements. There were also no significant differences in the analgesic and antiemetic requirements on POD 1, or in the length of hospital stay between the 2 groups.

**Table 4:**
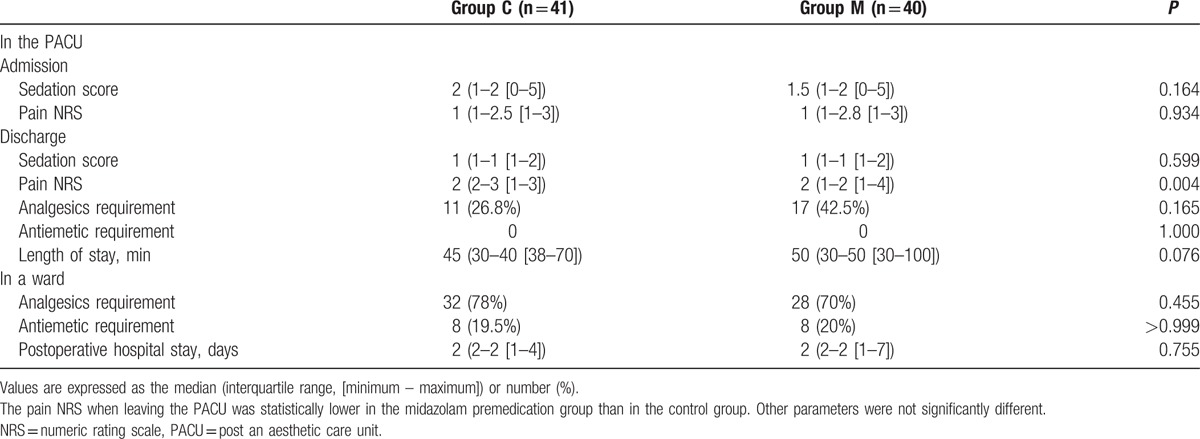
Postoperative recovery profiles.

## 4 Discussion

Our results demonstrated that midazolam premedication did not significantly affect patient-reported quality of recovery on POD 1 in female patients after mastectomy under general anesthesia with propofol–remifentanil. The dimensions of the QoR-40 subcomponent-scores also did not differ between patients receiving midazolam premedication or not.

All kinds of efforts for better quality of recovery should be performed to ensure the patients’ satisfaction and well-being. Better quality of recovery does not only rely on the postoperative analgesia and on postoperative nausea and vomiting control, but also on intraoperative treatments such as the appropriate anesthetic method or main anesthetic agents,^[15]^ addition of adjuvants to prevent postoperative complications,^[8,16–18]^ or adoption of new surgical procedures.^[19]^ Of course, such efforts should be planned for during the preoperative period already. Although some preoperative medication or treatments have been reported to be associated with an improvement in the quality of recovery by reducing postoperative pain,^[20,21]^ perioperative anxiety should not be overlooked in this aspect.

Preoperative anxiety has been reported to be associated with poor postoperative behavioral and clinical recoveries.^[22,23]^ Additionally, anxiety is the most common predictor for postoperative pain,^[24]^ and higher levels of preoperative anxiety are associated with an increased incidence of emesis.^[25]^ Intravenous midazolam as premedication has been shown to reduce the incidence of postoperative nausea, with a trend toward less postoperative vomiting for up to 24 hours postoperatively,^[7]^ and women administered intramuscular benzodiazepines 30 minutes before abdominal hysterectomy showed beneficial effects on postoperative analgesic requirements in 1 previous study,^[2]^ supporting the assumption that preoperative anxiolysis could lead to improved quality of recovery.

Moreover, a considerable degree of psychological distress might be expected especially in females undergoing breast cancer surgery, both due to the diagnosis and the anticipated alterations in body image.^[26,27]^ In addition, preoperative anxiety is generally maximized before transfer to the operating room.^[28]^ Therefore, premedication with anxiolytics targeting this moment in female patients undergoing mastectomy is thought to result in improved postoperative recovery.

However, the present study did not show any differential effects of midazolam premedication on the QoR-40 score. Several reasons may account for this result. First, postanesthetic recovery seems to be significantly influenced by the anesthesia method. TIVA with propofol and remifentanil has been shown to have superior effects to volatile anesthetics in terms of reduced postoperative nausea, vomiting, and pain.^[29,30]^ In our recent study, female patients undergoing thyroid surgery randomized to the TIVA group perceived a better quality of recovery on POD1 and POD 2 compared with patients in the desflurane group.^[15]^ Hence, the advantage of TIVA with propofol and remifentanil might conceal any beneficial effects of midazolam premedication.

The next consideration relates to the type of surgery. Mijderwijk and colleagues^[5]^ found that lorazepam, the most potent benzodiazepine, had no beneficial effects on the quality of recovery or on the manifestations of resistance in outpatients. These authors speculated that their result may have been related to the specific characteristics of outpatients and proposed that their study should be repeated in hospitalized patients undergoing major surgery who may be facing a longer stay in the hospital. However, even though the patients undergoing mastectomy are supposed to have a higher level of anxiety, mastectomy itself is not only a relatively minor surgery but also requires only a short in-hospital stay. Therefore, the physiologic disturbance immediately after surgery might not be significant in our study patients. Furthermore, in another previous study, compared with no premedication or placebo, the anxiolytic premedication with lorazepam did not enhance the self-reported patient experience the day after elective surgery with general anesthesia, but was related to the delayed time to extubation and decreased rate of cognitive recovery in the postoperative early phase.^[31]^ These findings suggest that long/intermediate-lasting anxiolytic medications, including benzodiazepines (e.g., lorazepam), should no longer be considered the standard premedication.

Lastly, there are various manifestations of preoperative anxiety. We considered that preoperative anxiety would reach its peak immediately before anesthesia induction and surgery. However, according to our interviews, many patients were more anxious about their cancer diagnosis than the surgery, and they often felt better postoperatively, owing to relief that the cancer had been removed. Consequently, pharmacologic premedication immediately before surgery might not be sufficient to relieve all anxiety and stress in these patients, and may hence also be insufficient to achieve better quality of recovery after anesthesia and surgery.

Of note, our study did not show any adverse effect from midazolam premedication, and the patients who received midazolam required smaller doses of the anesthetic agent and propofol and had shorter induction time compared with the subjects who received saline. These results are in agreement with previous reports, where premedication has been associated with a lower likelihood of the stormy induction of anesthesia associated with arterial desaturation.^[31]^ Furthermore, the anesthetic and analgesic doses can be modified according to the patients’ pain sensitivity and preoperative anxiety levels,^[32]^ and the addition of midazolam to a combination of opioids and propofol has been reported to be associated with a 71% reduction in effective site concentration.^[33]^

There are some limitations to the present study. First, it may be argued that a higher dose of midazolam might lead to deeper sedation and different results. However, a previous study demonstrated that a midazolam dose of 0.02 mg kg^−1^ was sufficient for producing sedation and anxiolysis with minimal effects on the cardiorespiratory status.^[3]^ On the other hand, a higher dose carries a higher risk of paradoxical reaction, hemodynamic changes, and respiratory depression^[34]^ and would not have been suitable for the aims of our study. Second, we only used a sedation scale in this study, rather than an anxiety scale such as the State-Trait Anxiety questionnaire.^[5]^ However, the anxiolytic effects of midazolam with this dose have been well documented already,^[1,4,5]^ and the main focus of our study was the functional recovery after surgery.

## 5 Conclusions

Our findings suggest that premedication with 0.02 mg kg^−1^ intravenous midazolam did not affect the postoperative QoR-40 recovery scores in female patients undergoing propofol-remifentanil anesthesia during in-hospital mastectomy. However, this premedication was associated with shorter induction times and lower total anesthetic consumption. More advanced studies, with more comprehensive anxiolytic regimens, administered during the entire preoperative period, and under more complex surgeries, are warranted in the future.
